# Diagnosis and management of human borreliosis in Romania

**DOI:** 10.1186/1756-3305-7-S1-P17

**Published:** 2014-04-01

**Authors:** P Mihailescu, D Predeteanu, A Craciunoiu, G Ceaus, C Istrate, C-M Cretu

**Affiliations:** 1 Eco-Para-Diagnostic - Parasitology Department, Bucharest, Romania; 2 University of Medicine and Pharmacy "Carol Davila"- Parasitology Department, Bucharest, Romania; 3 "Saint Mary" Hospital - Reumatology Department, Bucharest, Romania; 4 University Teaching Emergency Hospital - Neurology Department, Bucharest, Romania; 5 Colentina Teaching Hospital - Parasitology Department, Bucharest, Romania

## 

*Borrelia burgdorferi *sensu lato (s.l.) represents a category of bacteria, the etiologic agent of borreliosis, well known as Lyme Disease (LD). On our territory, *B. burgdorferi *s.l. is transmitted by the bite of infected ticks belonging to a few species of genus *Ixodes*. The most important species responsible for LD are *Borrelia burgdorferi *sensu stricto (s.s.)*, Borrelia afzelii *and *Borrelia garinii*.

The objectives of this study were to define the best way to confirm and consider Lyme disease using commercial serological tests: screening (indirect immunofluorescence test - IIFT) and confirmatory (DNA and Western Blotting - WB), according to the epidemiological, professional risks, animal contact and clinical signs/symptoms. The samples were collected from patients supposed with LD, referred to Eco-Para-Diagnostic Clinic, between October 2010 and December 2013.

A number of 1661 patients were referred to the clinic with LD suspicion and they were tested according to our protocol (serology, clinical, epidemiological). A number of 488 samples were tested using IIFT, 1086 with WB and 87 by DNA detection. Potential co-infections were also analyzed according to the history and clinical signs. Out of the 1661 suspected patients, 80 cases (study group) were confirmed by IIFT, WB, PCR, treated and monitored. Tick bite was recognized by 35 patients: 23 presented neurological symptoms, 10 cardiovascular signs, 20 rheumatologic signs, 12 dermatological signs (mostly EM) and one case presented ocular damage. The infection with a single *Borrelia burgdorferi *s.l. species was found in 56 cases, with two species in 22 cases and more than 2 species in 2 cases. Most common documented species (using the confirmation tests Western Blot and *Borrelia *DNA presence when it was possible): *Borrelia afzelii *(26) and *Borrelia burgdorferi *s.s. (19) and *Borrelia garinii (11)*. Confirmed co-infections (using IIFT/ELISA/WB techniques) included: *Bartonella quintana/henselae *(19), *Coxakie *virus (2), *Rickettsia *spp. (2), *Chlamydia pneumoniae *(11), *Mycoplasma pneumoniae *(10), *Ehrlichia *spp. (2), *Babesia *spp. (1), *Yersinia enterocolitica *(3). Co-morbidities were also evaluated for the differentials and the presence of multiple sclerosis, SLE, rheumatoid arthritis or ankylosing spondylitis was noticed. All patients received antibiotics, supplements and sugar restricted diet. Cases with confirmed associated diseases received concomitant specific therapy. Combinations of antibiotics were in accordance with LD and documented co-infections.

According to our results existence of LD in some parts of Romania was emphasized. The most common confirmed *Borrelia burgdorferi *s.l. species were *Borrelia afzelii *and *Borrelia burgdorferi *s.s. Early diagnosis and appropriate treatment are associated with a good evolution and prognosis. Chronic cases with multiple co-infections required longer treatment and monitoring. Multidisciplinary team works increase the accuracy of the diagnosis. Public health information and prophylactic measures should be correctly provided for the population, in different manners.

